# Statin treatment and LDL-cholesterol treatment goal attainment among individuals with familial hypercholesterolaemia in primary care

**DOI:** 10.1136/openhrt-2021-001817

**Published:** 2021-10-26

**Authors:** Barbara Iyen, Ralph K Akyea, Stephen Weng, Joe Kai, Nadeem Qureshi

**Affiliations:** Primary Care Stratified Medicine, Population Health and Lifespan Sciences, University of Nottingham Faculty of Medicine and Health Sciences, Nottingham, UK

**Keywords:** statins, primary care, epidemiology, electronic health records, hyperlipidaemia

## Abstract

**Objectives:**

Guidance recommends statin treatment in familial hypercholesterolaemia (FH) to achieve at least a 50% reduction in low-density lipoprotein cholesterol (LDL-C). We assessed statin prescribing rates and LDL-C treatment goal attainment among individuals with FH in primary care.

**Methods:**

Using primary care electronic health records from the UK Clinical Practice Research Datalink, we identified adults with recorded diagnosis of FH, statin treatment and measures of LDL-C prior to (baseline) and 12 months after initiating statin treatment. The percentage change in LDL-C was determined, and then baseline and treatment characteristics were assessed by LDL-C treatment goal attainment.

**Results:**

Of 3064 adults (mean age 50.8 years) with recorded diagnosis of FH and repeat LDL-C measures, 50% reduction in LDL-C from baseline was attained in 895 individuals (29.2%) in 12 months. Compared with those who did not attain this goal, these people were predominantly women; they were older at time of FH diagnosis (53.4 years vs 49.7 years) and first statin treatment (53.2 years vs 49.2 years) and had higher pretreatment total cholesterol (8.20 (SD 1.38) mmol/L vs 7.57 (SD 1.39) mmol/L) and pretreatment LDL-C (5.83 (SD 1.36) mmol/L vs 5.25 (SD 1.40) mmol/L). A higher proportion of individuals who attained the treatment goal was prescribed high-potency and medium-potency statins (24.3% and 71.7% vs 20.2% and 69.3%, respectively).

**Conclusions:**

Less than a third of individuals on statin treatment for FH in the community achieve recommended reductions in LDL-C. Greater awareness and optimisation of treatment for FH using higher-potency statins are needed.

**Key questions**

**What is already known about this subject?**

- The use of statins to reducing low-density lipoprotein cholesterol (LDL-C) burden is one of the central goals of familial hypercholesterolaemia (FH) management, and national and international guidance recommend that treatment should achieve at least a 50% reduction in LDL-C from baseline measurement.

**What does this study add?**

- This study provides novel evidence of suboptimal LDL-C reduction in individuals with FH who are being treated with statins, such that less than 40% of patients are treated with high-intensity statins at 12 months, and two-thirds of patients initiated on statin treatment fail to attain the recommended treatment target.

**How might this impact on clinical practice?**

- These findings highlight the need for greater awareness of FH and the need to optimally manage these patients with high-intensity statins.

## Introduction

Familial hypercholesterolaemia (FH) is a genetic condition that causes lifelong elevated low-density lipoprotein cholesterol (LDL-C) and is associated with significant cardiovascular morbidity and mortality.^1 2^ Reducing the LDL-C level is one of the primary goals in managing individuals with FH, and treatment with lipid-lowering drugs such as statins reduces LDL-C burden and consequently the cardiovascular disease (CVD) and mortality risk associated with FH.^3 4^

In the treatment of adults with FH, the UK National Institute for Health and Care Excellence (NICE) guidelines recommend at least a 50% reduction in serum LDL-C concentration from the baseline measurement.^5^ Recent guidelines from the European Society of Cardiology/European Atherosclerosis Society (ESC/EAS) recommend in addition to the LDL-C reduction of ≥50% from baseline, LDL-C treatment targets of <1.8 mmol/L in adults with FH, or <1.4 mmol/L in adults with FH and CVD or other major risk factors.^6^

The degree of LDL-C reduction varies with statins of different potencies.^7^ Also, there is interindividual variation in LDL-C reduction with statin therapy,^8^ which may partly be due to poor medication compliance^9^ or genetic factors.^10^ In the general population, it has been shown that over half of patients commenced on statin therapy for primary prevention of CVD do not attain optimal LDL-C reduction after 24 months of treatment.^11^ There is limited evidence of the magnitude of LDL-C reduction after initiation of statins in individuals with FH, in real-world community settings. This study explored statin prescribing patterns in individuals with FH in primary care and then assessed LDL-C reduction and treatment goal attainment in these patients following initiation of statins.

## Methods

### Data source

The UK Clinical Practice Research Datalink (CPRD) is a large electronic database of anonymised longitudinal routine primary care health records. The database has coverage of approximately 15% of the UK population, and patients are representative of the UK population in terms of age and sex.^12^ Individuals’ primary care records in CPRD were linked with their secondary care records (hospital episode statistics) and death registration records from the Office for National Statistics. Data access and ethical approval for the study were granted by the CPRD Independent Scientific Advisory Committee (protocol number 20_093) in June 2020.

### Study population

We identified all individuals aged 18 years or older who had a coded clinical diagnosis of FH in their primary care record, a record of treatment with statins of known potency, a measure of LDL-C prior to commencing statin treatment and a repeat LDL-C measure 12 months after initiating statin treatment. Patients were eligible for inclusion in the study if they had been registered in their general practice for at least 1 year and had no records of pre-existing CVD before being commenced on statins.

### LDL-C measures and other covariates

The baseline LDL-C measure was defined as the most recent LDL-C in patients’ records in the 12-month period before the first treatment with statins. LDL-C at 12 months was the last measure recorded after 6 months and within the 12 months after initiating statin treatment.

Patient characteristics collected at baseline include age; sex; ethnicity; history of alcohol misuse; smoking status; physical activity level; comorbidities including atrial fibrillation, hypertension, type 2 diabetes, overweight/obesity and chronic kidney disease; and use of medication that could secondarily cause hyperlipidaemia such as corticosteroids and antipsychotics. The categorising of statin potency (high, medium or low intensity) takes account of the estimated percentage reduction in LDL-C expected using varying doses of different statins.^13^ Low-intensity statins produce an LDL-C reduction of 20%–30%, medium-intensity statins produce a reduction of 31%–40% and high-intensity statins produce an LDL-C reduction greater than 40%^7^

### Outcome ascertainment

Patients were followed up from the first date of prescription of a statin with known potency. Baseline lipid measures (LDL-C) and repeated LDL-C measure at 12 months were used to determine the percentage change in LDL-C during the period of follow-up. Based on the NICE FH treatment guideline recommendation that states that statin treatment in individuals with FH should aim to achieve at least a 50% reduction in LDL-C concentration from baseline measurement,^14^ patients were classified as goal attainers or non–goal attainers, depending on whether they achieved the 50% or greater reduction in LDL-C or not, respectively.

### Statistical analyses

In the study cohort of individuals with FH, serum concentrations of lipids such as total cholesterol, LDL-C, high-density lipoprotein cholesterol and triglycerides, as well as types and potency of lipid-lowering treatment, were assessed at baseline, 12 months and 24 months. Change in LDL-C at 12 months was calculated by dividing the difference between the LDL-C at baseline and LDL-C at 12 months, by the baseline LDL-C concentration, and this was expressed as a percentage. As previously described, individuals were categorised as goal attainers or non–goal attainers, depending on their percentage change in LDL-C at 12 months. Baseline characteristics including the clinical profile and treatment characteristics were explored in the cohort who attained the 50% LDL-C reduction treatment goal and those who did not. χ^2^ test of significance, t-tests and Mann-Whitney U tests were used to assess differences in categorical and continuous variables between LDL-C goal attainers and non–goal attainers, depending on the distribution of the variables. After determining the change in LDL-C at 12 months, further analyses determined change in LDL-C at 24 months. LDL-C at 24 months was defined as the last LDL-C measure after 12 months but within the 24-month period after initiating statin treatment. We further assessed attainment of at least a 40% reduction in LDL-C, which is the NICE-recommended target for the general population of non-FH individuals who are initiated on statins for the primary prevention of CVD.^15^

Following primary analyses conducted on all individuals with FH diagnosis in primary care, a sensitivity analysis was done on the subset of individuals who had a diagnosis of FH as well as baseline total cholesterol of 7.5 mmol/L or greater, which is the FH total cholesterol diagnostic threshold.^2^ Further sensitivity analysis also assessed treatment goal attainment with respect to the ESC/EAS guidelines.^6^ Statistical significance was defined at the p<0.05 level. Analyses were conducted using Stata SE V.16.

## Results

A total of 3064 adults had clinical diagnostic codes for FH, records of statin with known potency at baseline, LDL-C measures at baseline and 12 months and no history of CVD prior to being initiated on statins, between December 1988 and August 2020. Statin treatment and lipid profile of these individuals at baseline, as well as 12 and 24 months after starting treatment with statins, are shown in table 1.

**Table 1:** Lipid profile, statin treatment and treatment goal attainment in adults with FH in UK primary care (n=3064)

Characteristics	Unit	Baseline	12 months	24 months
Individuals’ lipid profile				
Total cholesterol (mmol/L)	Mean (SD)	7.76 (1.42)	5.56 (1.40)	5.42 (1.30)
LDL-cholesterol (mmol/L)	Mean (SD)	5.42 (1.41)	3.45 (1.37)	3.28 (1.23)
HDL-cholesterol (mmol/L)	Mean (SD)	1.50 (0.49)	1.49 (0.47)	1.51 (0.49)
Triglycerides (mmol/L)	Median (IQR)	1.70 (1.20–2.47)	1.40 (1.00–2.00)	1.32 (0.93–2.00)
Potency of prescribed statins*				
		(n=3064)	(n=2142)	(n=1940)
Low	n (%)	264 (8.62)	142 (6.63)	111 (5.72)
Medium		2145 (70.01)	1225 (57.19)	1067 (55.00)
High		655 (21.38)	775 (36.18)	762 (39.28)
LDL-C reduction at follow-up				
FH treatment goal attainment			(n=3064)	(n=1662)
Attained ≥50% reduction	n (%)		895 (29.21)	558 (33.57)
Non-attainment of 50% reduction			2169 (70.79)	1104 (66.43)
General population goal attainment			(n=3064)	(n=1662)
Attained ≥40% reduction	n (%)		1566 (51.11)	935 (56.26)
Non-attainment of 40% reduction			1498 (48.89)	727 (43.74)

*Statin potency at 12 months and 24 months was reported in individuals with LDL-C records at baseline and 12 months, as well as statin potency records at 12 and 24 months, respectively. Total number and percentage n (%) for potency were based only on those with potency records, with the exclusion of missing records.

FH, familial hypercholesterolaemia; HDL, high-density lipoprotein; LDL, low-density lipoprotein.

Over the period of follow-up, the proportion of individuals prescribed with low-potency statins reduced from baseline to 24 months (8.6% at baseline, 6.6% at 12 months and 5.7% at 24 months), while the proportion prescribed with high-potency statins increased over time, from 21.4% at baseline, 36.2% at 12 months and 39.3% at 24 months. Compared with the baseline LDL-C value, there was a mean reduction in LDL-C of 36.3% at 12 months (5.42 mmol/L vs 3.45 mmol/L) and 39.5% at 24 months (5.42 mmol/L vs 3.28 mmol/L).

Prior to restricting our study sample to the 3064 eligible adults with FH (who were treated with statins, with no CVD prior to statin initiation and with LDL-C measures at baseline and 12 months), we assessed the pattern of lipid-lowering treatment prescribing 8234 adults with FH and no prior CVD. Findings showed an increase in the prescribing of non-statin lipid-lowering treatments such as ezetimibe (monotherapy or prescribed in combination with statins) (0.05% at baseline, 3.2% at 12 months and 4.2% at 24 months) and fibrates (0% at baseline, 1.1% at 12 months and 1.4% at 24 months) (online supplemental table 1).

10.1136/openhrt-2021-001817.supp1 Supplementary data

### LDL-C treatment goal attainment

Of the 3064 individuals in our FH study cohort, 895 (29.2%) achieved a 50% or greater reduction in LDL-C concentration from the baseline measure, 12 months after statin initiation. Repeat LDL-C measures were available for 1662 individuals at 24 months, and 558 (33.6%) achieved a 50% or greater reduction in LDL-C from baseline (table 1).

A 40% reduction in LDC-C from baseline, which is the NICE-recommended goal for the general population of non-FH individuals initiated on statins for primary prevention of CVD, was achieved at 12 months by 51% of the FH cohort and at 24 months by 56.3% of the cohort.

### Characteristics of individuals by LDL treatment goal attainment at 12 months

**Figure 1:**
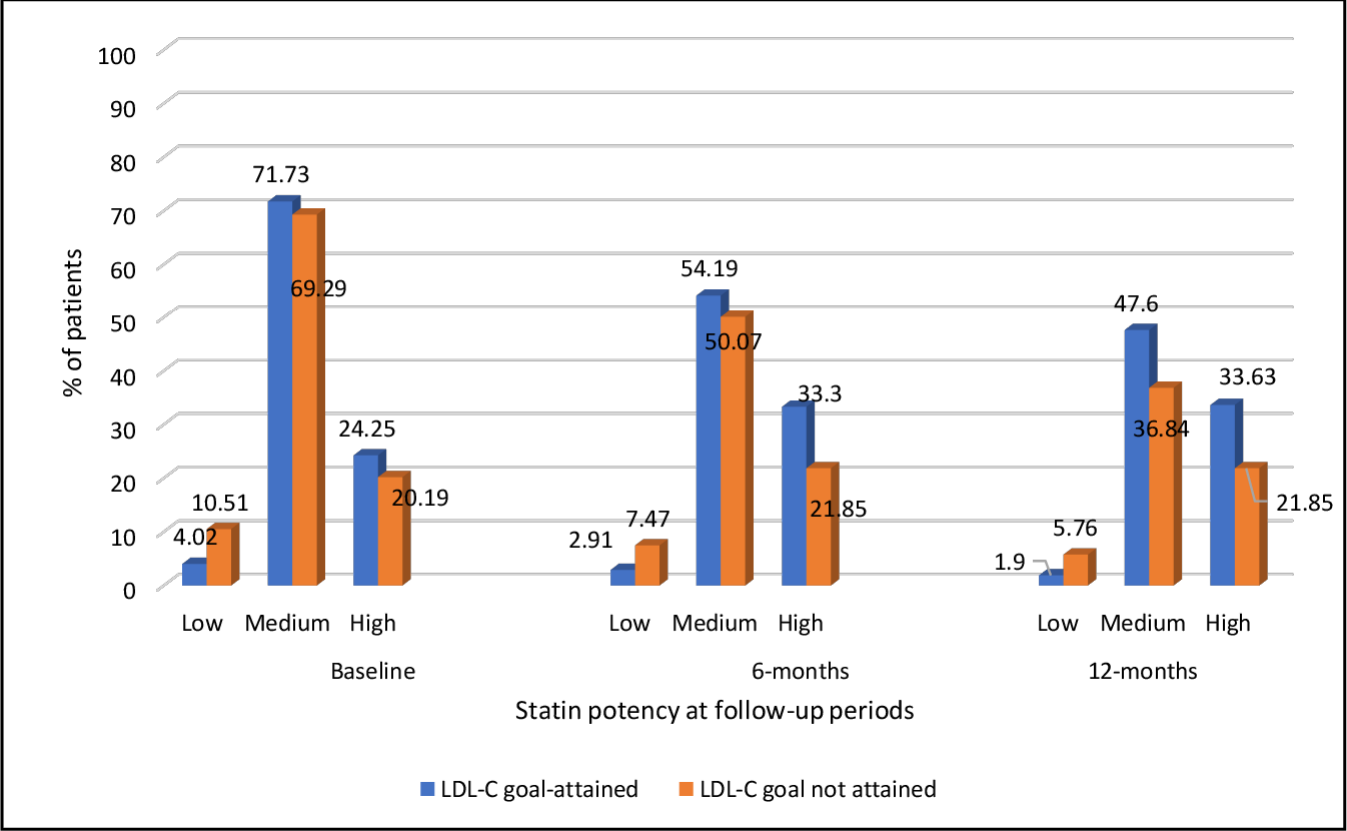
Prescribing of low-potency, medium-potency and high-potency statins among LDL-C goal attainers and non–goal attainers, at time of statin initiation (baseline) and 6 months and 12 months after statin initiation. LDL-C, low-density lipoprotein cholesterol.

Table 2 shows the baseline and lipid-lowering treatment characteristics of individuals, by LDL treatment goal attainment at 12 months. Compared with individuals who did not attain the LDL-C treatment goal of 50% or greater, a significantly higher proportion of those who attained the goal was women. Individuals who attained the treatment goal were of older age at time of FH diagnosis (53.4 years vs 49.7 years) and at time of first statin treatment (53.2 years vs 49.2 years), and they had significantly higher mean pretreatment total cholesterol (8.20 (SD 1.38) mmol/L vs 7.57 (SD 1.39) mmol/L) and pretreatment LDL-C (5.83 (SD 1.36) mmol/L vs 5.25 (SD 1.40) mmol/L) than those who did not attain the treatment goal. Overall, the proportion of individuals treated with low-intensity and medium-intensity statins reduced, while the proportion of prescribed with high-intensity statins increased from baseline to 12 months. Significantly more people who attained the LDL-C treatment goal were treated with medium-intensity and high-intensity statins than those who did not attain the treatment goal, at baseline (71.7% and 24.3% vs 69.3% and 20.2%, respectively), 6 months (54.2% and 33.3% vs 50.1% and 21.9%, respectively) and 12 months (47.6% and 33.6% vs 36.8% and 21.9%, respectively) after statin initiation (table 2 and figure 1). Compared with individuals who attained the treatment goal, significantly higher proportions of those who did not attain the treatment goal were smokers at the time of their first statin treatment (31.0% vs 26.0%). There were no significant differences in ethnicity, mean pretreatment triglycerides, alcohol consumption and prevalence of comorbidities such as atrial fibrillation, hypertension, type 2 diabetes, obesity and physical activity levels between those who attained a ≥50% LDL-C target and those who did not. The prevalence of chronic kidney disease was higher in those who attained the treatment goal compared with those who did not (2.1% vs 1.0%), and more people who attained the treatment goal were on antipsychotics and corticosteroids than in those who did attain the treatment goal.

**Table 2:** Baseline and treatment characteristics of individuals, by LDL-cholesterol treatment goal attainment at 12 months (n=3064)

Patient or treatment characteristics	Unit	Total N (%)	LDL-C goal attained N (%)	LDL-C goal not attained N (%)	P value
		3064 (100)	895 (29.21)	2169 (70.79)	
Women	N (%)	1873 (61.13)	590 (65.92)	1283 (59.15)	<0.001
Ethnicity	N (%)				
White		1610 (91.74)	452 (92.24)	1158 (91.54)	
Asian/British Asian		74 (4.22)	10 (4.08)	54 (4.27)	0.366
Black/Black British		27 (1.54)	7 (1.43)	20 (1.58)	
Mixed		10 (0.57)	0	10 (0.79)	
Others		34 (1.94)	11 (2.24)	23 (1.83)	
Age (years) at FH diagnosis	Mean (SD)	50.79 (13.13)	53.44 (12.60)	49.71 (13.20)	<0.001
Age (years) at first statin	Mean (SD)	50.33 (12.83)	53.18 (12.48)	49.16 (12.80)	<0.001
Pretreatment total cholesterol (mmol/L)	Mean (SD)	7.76 (1.42)	8.20 (1.38)	7.57 (1.39)	<0.001
Pretreatment LDL-C (mmol/L)	Mean (SD)	5.42 (1.41)	5.83 (1.36)	5.25 (1.40)	<0.001
Post-treatment LDL-C (12 months) (mmol/L)	Mean (SD)	3.45 (1.37)	2.43 (0.63)	3.88 (1.37)	<0.001
Pretreatment HDL-C (mmol/L)	Mean (SD)	1.50 (0.49)	1.59 (0.57)	1.46 (0.45)	<0.001
Pretreatment triglycerides (mmol/L)	Mean (SD)	1.70 (1.20–2.47)	1.70 (1.20–2.47)	1.70 (1.20–2.46)	0.5087
Statin potency at baseline	N (%)				
Low		264 (8.62)	36 (4.02)	225 (10.51)	
Medium		2145 (70.01)	642 (71.73)	1503 (69.29)	<0.001
High		655 (21.38)	217 (24.25)	438 (20.19)	
Statin potency at 6 months	N (%)				
Low		188 (6.14)	26 (2.91)	162 (7.47)	
Medium		1571 (51.27)	485 (54.19)	1086 (50.07)	<0.0001
High		772 (25.20)	298 (33.30)	474 (21.85)	
Missing record		533 (17.40)	86 (9.61)	447 (20.61)	
Statin potency at 12 months	N (%)				
Low		142 (4.63)	17 (1.90)	125 (5.76)	<0.0001
Medium		1225 (39.98)	426 (47.60)	799 (36.84)	
High		775 (25.29)	301 (33.63)	474 (21.85)	
Missing record		922 (30.09)	151 (16.87)	771 (35.55)	
Cigarette smoking status at baseline	N (%)	n=2126	n=615	n=1511	
Current		628 (29.54)	160 (26.02)	468 (30.97)	<0.001
Ex		461 (21.68)	135 (21.95)	326 (21.58)	
Never		1037 (48.78)	320 (52.03)	717 (47.45)	
Alcohol misuse (yes)	N (%)	35 (1.14)	14 (1.56)	21 (0.97)	0.158
Atrial fibrillation	N (%)	18 (0.59)	8 (0.89)	10 (0.46)	0.154
Chronic kidney disease	N (%)	40 (1.31)	19 (2.12)	21 (0.97)	0.01
Hypertension	N (%)	406 (13.25)	120 (13.41)	286 (13.19)	0.869
Type 2 diabetes	N (%)	45 (1.47)	15 (1.68)	30 (1.38)	0.54
Obesity/overweight	N (%)	320 (10.44)	97 (10.84)	223 (10.28)	0.647
Physical activity level	N (%)	n=331	n=94	n=237	
Extremely inactive		23 (6.95)	7 (7.45)	16 (6.75)	0.54
Sedentary		29 (8.76)	7 (7.45)	22 (9.28)	
Moderately active		271 (81.87)	76 (80.85)	195 (82.28)	
Extremely active		8 (2.42)	4 (4.26)	4 (1.69)	
Use of other medications	N (%)				
Antipsychotics		418 (13.64)	148 (16.54)	270 (12.45)	0.003
Corticosteroids		292 (9.53)	108 (12.07)	184 (8.48)	0.002

FH, familial hypercholesterolaemia; HDL, high-density lipoprotein; LDL, low-density lipoprotein.

### Sensitivity analyses

Table 3 shows the result of analyses restricted to 1805 individuals who had clinical FH diagnoses and baseline total cholesterol of 7.5 mmol/L or greater. Higher proportions of these individuals were treated with high-potency statins at baseline and 12 and 24 months, compared with the entire study cohort. Compared with the LDL-C concentration at baseline, there was a mean reduction in LDL-C of 39.8% at 12 months (6.16 mmol/L vs 3.71 mmol/L) and 43.0% at 24 months (6.16 mmol/L vs 3.51 mmol/L), which were greater than reductions observed in the entire cohort. Also, compared with the entire study cohort, a greater proportion of these individuals attained the NICE guideline–recommended LDL-C reduction of 50% or greater at 12 months (36.0% vs 29.2%) and 24 months (43.1% vs 33.6%).

Lastly, assessment of the LDL-C treatment goal attainment with respect to the ESC/EAS guidelines showed that only 4.5% of the study cohort achieved the goal of 50% LDL-C reduction and LDL-C <1.8 mmol/L (online supplemental table 2). Further restrictions to the subset of individuals with clinical FH diagnoses and a diagnostic threshold of total cholesterol ≥7.5 mmol/L did not demonstrate an increase in the proportions who attained the ESC/EAS treatment goals.

**Table 3:** Lipid profile at baseline, lipid-lowering treatment and treatment goal attainment, in sensitivity analyses restricted to adults with FH diagnosis and baseline total cholesterol ≥7.5 mmo/L (n=1805 at baseline)

		Baseline	12 months	24 months
Total cholesterol (mmol/L)	Mean (SD)	8.62 (1.09)	5.87 (1.47)	5.68 (1.33)
LDL-cholesterol (mmol/L)	Mean (SD)	6.16 (1.25)	3.71 (1.45)	3.51 (1.29)
HDL-cholesterol (mmol/L)	Mean (SD)	1.53 (0.49)	1.52 (0.48)	1.53 (0.46)
Triglycerides (mmol/L)	Median (IQR)	1.80 (1.29–2.65)	1.40 (1.00–2.10)	1.39 (1.00–2.00)
Potency of prescribed statins*	N (%)	(n=1805)	(n=1299)	(n=1172)
Low		144 (7.98)	84 (6.47)	56 (4.78)
Medium		1231 (68.20)	668 (51.42)	572 (48.81)
High		430 (23.82)	547 (42.11)	544 (46.42)
LDL-C reduction at time of follow-up	N (%)		(n=1805)	(n=982)
Attained 50% reduction			649 (35.96)	423 (43.08)
Non-attained 50% reduction			1156 (64.04)	559 (56.92)

*Statin potency at 12 months and 24 months was reported in individuals with records of LDL-C at 12 months, as well as records of statin potency at 12 and 24 months, respectively.

FH, familial hypercholesterolaemia; HDL, high-density lipoprotein; LDL, low-density lipoprotein.

## Discussion

In this large prospective cohort of adults with FH, the NICE-recommended treatment goal of at least a 50% reduction in LDL-C from baseline was achieved in less than a third of individuals treated with statins. The observed mean reduction in LDL-C from baseline was 36.3% at 12 months and 39.5% at 24 months following the initiation of statin therapy. Compared with those who did not achieve the treatment target, individuals who achieved the treatment target of ≥50% reduction in LDL-C from baseline were predominantly women, of older age at time of FH diagnosis and on initiation of statin treatment; they had more severe mean pretreatment LDL-hypercholesterolaemia, and significantly higher proportions of them was treated with high-potency statins at baseline and over the 12-month period after the initiation of statins. Individuals with FH diagnoses, who additionally had baseline LDL-C levels at or above the FH diagnostic threshold of 7.5 mmol/L, had greater 12-month and 24-month reductions in LDL-C after initiating statins, and a higher proportion of them attained the NICE guideline LDL-C treatment goal at 12 and 24 months compared with the entire cohort.

### Strengths and limitations

To our knowledge, this is the first study to assess and quantify LDL-C reduction associated with statin treatment in individuals with FH in primary care. By using pseudonymised but comprehensively coded electronic primary care health records from a quality-assured and highly representative database,^12^ we were able to ascertain repeated measures of LDC-C and robustly characterise these individuals using a large sample size and a longitudinal study design. The study design minimised selection, recall and respondent bias.

We recognise certain limitations. As with all primary care electronic health records, the recording of clinical diagnosis relies on the accurate and complete recording by the general practitioner (GP) during routine consultations.^16 17^ While we acknowledge the potential for FH misclassification in some individuals with hypercholesterolaemia due to secondary causes, there were no significant differences in the prevalence of comorbid conditions between those who attained and those who did not attain the LDL-C treatment goal, and thus, there is no reason to believe differential effects of such misclassification between the groups. It was not possible to determine whether recorded FH diagnoses were made following clinical assessment of FH phenotype or specialist assessment including genetic testing in secondary care or both, because there are currently no distinct codes for these in clinical systems. As there was no data on FH genetic mutation, it was not possible to assess whether there was an association between the various FH genetic mutation types and LDL-C treatment goal attainment. Lastly, although we explored individuals’ sociodemographic and clinical characteristics in relation to their LDL-C goal attainment or non-attainment status, we did not have data on some of the factors that may be associated with treatment goal attainment such as side effects to statin treatment, non-adherence to treatment and dietary intake, and so, we were unable to explore these in our FH study cohort. Despite the above limitations, findings from our study reflect a pragmatic evaluation of LDL-C treatment goal attainment associated with statin therapy in primary care patients with FH.

### Comparison with previous literature

This is the first study to evaluate LDL-C response to statin treatment in primary care patients with FH. Suboptimal response to statins had previously been demonstrated in over half of the general population of people initiated on statins for primary prevention.^11^ Similarly, findings from the EURO ASPIRE V Survey in 78 centres from 16 European countries found that the recommended LDL-C target was achieved in less than half of high–CVD risk individuals on lipid-lowering medication.^18^ In the FH population, previous studies of lipid-lowering therapy have primarily evaluated treatments among patients in lipid clinics or specialist registers,^19–21^ while clinical trials have assessed LDL-C reduction associated with treatment using more specialised lipid-lowering agents such as proprotein convertase subtilisin/kexin type 9.^22 23^ While it had previously been shown that less than 50% of the general population who was prescribed statins for primary CVD prevention achieved a 40% or greater reduction in LDL-C within 24 months, our study of the FH population found that 51.1% of individuals at 1 year and 56.3% of individuals at 2 years achieved this general population treatment target. This suggests that although individuals with FH may be treated better with statins than the general population, their lipid-lowering management is not optimal to the level recommended in the FH guidelines. The importance of intensive GP management of FH has been demonstrated in a study by Brett *et al*, which showed that implementing a pragmatic intervention plan by GPs, with the inclusion of statin/ezetimibe medication±lifestyle advice, resulted in significant reduction in LDL-C levels.^24^

While there is no clear explanation for the variation in LDL-C reduction in our study population, we hypothesise that this may be partly explained by the significant differences in baseline and treatment characteristics between individuals who attained the LDL-C treatment goal and those who did not. Perhaps older adults and those with higher LDL-C at baseline are more likely to be prescribed higher-intensity statins due to their perceived increased risk of CVD, which consequently results in better LDL-C treatment outcomes.

There were no significant differences in alcohol consumption or the reported level of physical activity between statin treatment goal attainers and non-attainers, but cigarette smoke exposure was higher in those who failed to attain the LDL-C treatment goal. Although there is no clear evidence that smoking increases the levels of LDL-C,^25^ exposure to cigarette smoke is likely to further increase CVD risk in these individuals who fail to attain the LDL-C treatment goal.

### Clinical implications and conclusion

This study provides novel knowledge of suboptimal LDL-C reduction in individuals with FH who are being treated with statins. With less than 40% of patients being treated with high-intensity statins and lack of attainment of the ≥50% LDL-C-lowering treatment goal in two-thirds of patients initiated on statin treatment, findings from this study highlight significant gaps in the FH patient management. Applying multifaceted approach by identifying the barriers to evidence-based FH patient care and using a strategic means to tailor effective interventions to modifiable barriers at the patient level, physician level and health system level are likely to improve implementation of evidence-based FH patient care and outcomes.^26 27^ Emphasis on lifestyle and dietary modification is needed to manage raised LDL-C and reduce CVD risk in individuals with FH. Intensive LDL-C lowering has clear benefits in CVD risk reduction,^28 29^ so it is essential that these individuals in primary care are optimally managed and intensively monitored. Raising greater awareness of FH, in particular of the need for use of higher-potency statins, is needed in this setting.

## Data Availability

Data are available on reasonable request. The CPRD data analysed during this study are available from the Clinical Practice Research Datalink (CPRD) (enquiries@cprd.com), but restrictions apply to the availability of these data, which were used under license for the current study and so are not publicly available. Data are however available from the authors on reasonable request and with permission of the CPRD Independent Scientific Advisory Committee (ISAC) (enquiries@cprd.com).
