# Supplementary material for: Statin treatment and LDL-cholesterol treatment goal attainment among individuals with familial hypercholesterolaemia in primary care
Source: Open Heart. 2021 Oct 26;8(2):e001817. doi: 10.1136/openhrt-2021-001817 (PMC8549660; doi:10.1136/openhrt-2021-001817)
Supplement: Supplementary data [file openhrt-2021-001817supp001.pdf]

Supplementary Tables

Supplementary table 1. Lipid-lowering treatment among individuals with FH in UK Primary Care (n=8,234) *

		Baseline	12 months	24 months
**Type of lipid-lowering treatment**				
Statins		8,137 (98.82)	5,944 (72.19)	5,341 (64.87)
Ezetimibe + Statins	n (%)	4 (0.05)	8 (0.10)	13 (0.16)
Fibrates		-	87 (1.06)	115 (1.40)
Lipid-regulating drugs (Ezetimibe)		-	258 (3.13)	329 (4.00)
Unknown		93 (1.13)	1,937 (23.53)	2,436 (25.59)

*Table shows data from analyses of individuals wit FH prior to applying study eligibility criteria

Supplementary table 2. Attainment of European Society of Cardiology/ European Atherosclerosis Society (ESC/EAS) guideline of 50% LDL-C reduction and LDL-C <1.8mmol/L

EAS treatment goal attainment in study population		12 months	24 months
**Goal attainment in entire study cohort**		(n=3,064)	(n=1,662)
Attained 50% LDL-C & LDL-C <1.8mmol/l	n (%)	139 (4.5)	81 (4.87)
Goal not attained		2,925 (95.46)	1,581 (95.13)
Goal attainment in FH cohort with baseline LDL>=7.5		(n=1,805)	(n=982)
Attained 50% LDL-C & LDL-C <1.8mmol/l	(%)	51 (2.83)	36 (3.67)
Goal not attained		1,754 (97.17)	946 (96.33)
